# Growth optimization and identification of an ω-transaminase by a novel native PAGE activity staining method in a *Bacillus* sp. strain BaH isolated from Iranian soil

**DOI:** 10.1186/s13568-021-01207-7

**Published:** 2021-03-23

**Authors:** Najme Gord Noshahri, Jamshid Fooladi, Ulrike Engel, Delphine Muller, Michaela Kugel, Pascal Gorenflo, Christoph Syldatk, Jens Rudat

**Affiliations:** 1 Department of Biotechnology, Faculty of Biology Science, Alzahra University, Tehran, Iran; 2 Karlsruhe Institute of Technology (KIT), BLT 2 Technical Biology, Fritz-Haber-Weg 4, Karlsruhe, Germany

**Keywords:** ω-Transaminase, *ortho*-Xylylenediamine assay, Native PAGE, Activity staining, Growth optimization, *Bacillus* fermentation

## Abstract

ω-Transaminases’ (ω-TAs) importance for synthesizing chiral amines led to the development of different methods to quickly identify and characterize new sources of these enzymes. Here we describe the optimization of growth and induction of such an enzyme in a wild type strain of *Bacillus* sp. strain BaH (IBRC-M 11337) isolated from Iranian soil in shaking flasks by the response surface methodology (RSM). Optimum conditions were set in a multiplexed bench-top bioreactor system (Sixfors). ω-TA activity of obtained biomass was checked by an innovative efficient colorimetric assay for localizing ω-TAs in crude extracts on acrylamide gel by using *ortho*-xylylenediamine (OXD) as amino donor. The application of the established OXD assay is thereby expanded from high-throughput activity screenings and colony-based screenings of heterologously expressed mutants to a direct identification of ω-TAs in wild-type strains: This assay can be used to detect the protein band of the respective enzyme in crude extracts of novel isolates by visual inspection of native PAGEs without any upstream protein purification, thus enabling subsequent further investigations of a newly discovered enzyme directly from the crude extract.

**Supplementary Information:**

The online version contains supplementary material available at 10.1186/s13568-021-01207-7.

## Introduction

Discovery and characterization of new ω-transaminases (ω-TAs) to synthesize pure chiral amines has drawn attention (Gomm et al. 2016) as an alternative green approach instead of chemical synthesis via transition-metal catalysis (Weiß et al. 2017). Enantiopure amines play an important role as building blocks in the production of pharmaceuticals and fine chemicals (Höhne and Bornscheuer 2009). ω-TAs are members of the pyridoxal 5ʹ-phosphate (PLP)-depending enzyme family that transfers amine groups from amine donors to aldehydes or ketones as amine acceptor. An ω-TA catalyzed synthesis of chiral amines can be performed via kinetic resolution of racemic amines or by asymmetric synthesis where the amine group of an amine donor is transferred to ketones or aldehydes with usually high enantioselectivity (Weiß et al. 2017).

**Fig. 1:**
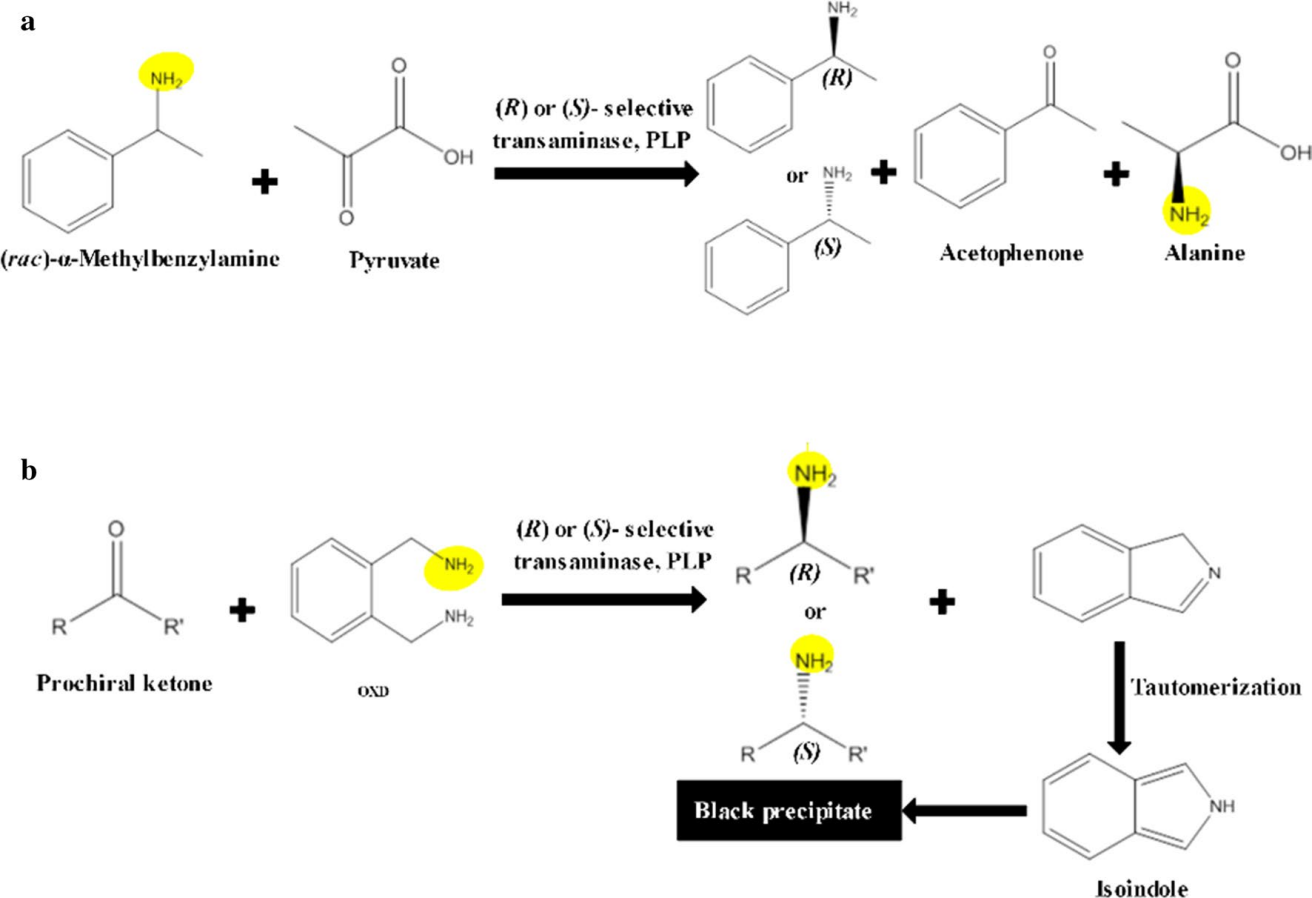
ω-TA activity towards different substrates. **a** ω-TA catalyzed reaction of (*rac*)-MBA with α-keto acid such as pyruvate. **b** Color developing process in ω-TA reaction by using *ortho*-xylylenediamine (OXD) as amine donor. R and R^’^ refer to the residues defined by the substrate specifity of the applied enzyme, amine group was highlighted to show how amine group is stereospecifically transferred to the ketone by ω-TA activity

As the expression of ω-TAs is growth-associated and essential in nitrogen metabolism, nitrogen plays an important role in the induction of these enzymes (Shin and Kim 2001). (*rac*)-α-methylbenzylamine (MBA) and *o*-xylylenediamine (OXD) are promising amine donors to survey ω-TA activity (Buß et al. 2018c; Shin and Kim 2001). The former is a model amine donor (Shin and Kim 1997) which is converted to acetophenone (AcPhe) after transformation of amine group to ketone during ω-TA activity, then, AcPhe concentration can be quantified (Guo and Berglund 2017) (Fig. 1a). The latter was originally reported by Green et al. (2014): OXD reaction is irreversible since after amine transfer the molecule spontaneously cyclizes and tautomerizes to the stable isoindole product which leads to the generation of an insoluble black polymer by oxidative processes (Fig. 1b). OXD is a substrate for most (*R*)- and (*S*)*-*selective ω-TAs that have been assayed so far and can be used as amine donor in combination with a plethora of amine acceptors as demonstrated by Kelly (Kelly et al. 2017). The OXD colorimetric assay can be applied to high-throughput screening of ω-TAs in liquid phase (Buß et al. 2018c) or recombinant colonies expressing transaminase in solid phase (Green and Turner 2016).

Our recently published research on screening wild-type bacterial strains exhibiting ω-TA activities led to the identification of promising *Bacillus* strains from Iranian soil by using (*rac*)-α-MBA as sole nitrogen source in the medium. Among them, *Bacillus* sp. strain BaH (IBRC-M 11337) exhibited ω-TA activity towards several amine donors and acceptors with broad pH tolerance from 5 to 9 (Gord Noshahri et al. 2019).

The highest potential of ω-TA activity of *Bacillus* sp. strain BaH (BaH-ω-TA) against bulky substrates encouraged us to increase its production in the wild-type strain by growth optimization and enzyme induction using the response surface methodology (RSM).

Although many ω-TAs from a plethora of microorganisms have been described to be produced recombinantly, there are a few reports to cultivate the related wild-type strains in bioreactors which can be important for enzymes hard to express heterologously, e.g. from extremophile microorganisms. Buß et al. (2018a) studied β-phenylalanine degradation during fermentation of *Paraburkholderia*. Cultivation of *Arthrobacter* sp. KNK168 in a 5 L fermenter was reported by Iwasaki et al. (2006); both publications, however, do not describe any attempts for growth optimization.

Designing a fermentation system in a bioreactor depends on knowledge of several reaction parameters on cultivation such as pH, temperature, dissolved oxygen (DO) concentration and agitation speed (Hsu and Wu 2002). In case of enzyme production, confirmation of enzyme activity is a necessary step during fermentation.

In this study, the optimization of cell growth of *Bacillus* sp. strain BaH along analyzing ω-TA activity in shaking flasks and subsequently in a multiplexed bench-top fermenter system was investigated. Enzyme activity was confirmed by a colorimetric assay on acrylamide gel and kinetic parameters were determined.

## Materials and methods

### Chemicals

Otherwise indicated, all chemicals and solvents were purchased from Sigma-Aldrich (St. Louis, MO, US) and Carl Roth GmbH (Karlsruhe, Germany).

### Bacterial strains and culture media

ω-TA mutant with PDB code 3FCR of *Ruegeria* sp. TM1040 was kindly provided by Uwe Bornscheuer (Greifswald/Germany) (Buß et al. 2018c). *Bacillus* sp. strain BaH (IBRC-M 11337) was isolated from Iranian soil as described by Gord Noshahri et al. (2019) and is preserved by IBRC (Iranian Biological Resource Center). Expression of the ω-TA mutant 3FCR of *Ruegeria* sp. TM1040 in *E. coli* BL21 was conducted according to Buß et al. (2018b). *Bacillus* sp. strain BaH was grown in minimal medium (MIM) containing 18 mM (*rac*)-α-methylbenzylamine (MBA) and 100 mM glycerol as sole nitrogen and carbon sources and salts including 1 g/L MgSO_4_·7H_2_O, 4 mg/L FeSO_4_·7H_2_O, 0.02 mg/L H_3_BO_3_, 0.1 mg/L ZnCl_2_, 0.2 mM CaCl_2_, 0.05 CoCl_2_, 0.1 mg/L MnSO_4_·4H_2_O, 0.05 mg/L CoCl_2_, 0.1 mg/L CuSO_4_·5H_2_O, 2.0 mg/L NaMoO_4_, 0.1 mg/L NiSO_4_ · 6H_2_O in potassium phosphate buffer (50 mM) (Yun et al. 2004). FeSO_4_ and (*rac*)-MBA were separately filtrated for sterilization and added to the medium after autoclaving.

### Effect of α-MBA on ω-TA induction

A single colony of *Bacillus* sp. strain BaH was cultured separately in 25 ml LB and MIM media in shaking flask (100 mL) by incubation at 35 °C with 120 rpm until the absorbance at 600 nm (OD_600_) reached approximately 2.0. The cells were harvested by centrifugation (6000×*g*, 10 min). Then the cell OD_600_ was adjusted to ~ 20 by resuspension in HEPES buffer (50mM, pH 7.5). ω-TA activity was surveyed by using 50 µL cell suspension in a 150 µL reaction mixture composed of 10 % dimethyl sulfoxide (DMSO), 1 mM PLP in HEPES buffer (50mM, pH 7.5). 7.5mM *o*-xylylenediamine (OXD), and 5 mM pyruvate were added to reaction as an amine donor and amine acceptor, respectively (Buß et al. 2018c). The reactions were carried out in 96-well plate and incubated for five hours at 35 °C with 150 rpm. 3FCR cells grown in auto-inducing medium (Formedium, UK) as described by Buß et al. (2018b) were used as a positive control. Each reaction was done in duplicate. HEPES buffer without cells was used as negative control.

### Optimization with response surface methodology (RSM)

To determine the effect of three variables including temperature, pH, and agitation speed on biomass production by *Bacillus* sp. strain BaH was survey by RSM. It is a statistical method which investigates combined effects of independent variables (Sunitha et al. 1999) and has been applied in various studies (Ahmad and Panda 2014; Singh et al. 2011; Sunitha et al. 1999). Experimental designing was formulated according to central composite design (CCD) by using Design Expert 7.0.0 software (Stat-Ease Inc., USA). The three factors: temperature, pH, and agitation speed were surveyed in three levels (+), (0) and (−) with three repetitions (Table 1). In total, 15 runs were generated with central point: temperature 33 °C, pH 7.7, 160 rpm agitation. Table 1 shows coded levels for independent variables. Biomass production (OD_600nm)_ and AcPhe formation (deaminated form of MBA) were considered as responses. Point prediction of the Design Expert was applied to obtain optimum value of the factors for production maximum level of biomass. Then, predicted condition was run with triplicate experiments to survey model accuracy.

**Table 1:** Level of factors used in experimental design

Factors	Symbol	Coded-variable level		
		− 1	0	1
Temperature (°C)	A	26	33	40
Agitation speed (rpm)	B	120	160	216
pH	C	6.7	7.7	8.5

### Growth optimization in shaking flasks

All designed experiments were applied in 500 mL baffled shaking flasks containing 100 mL medium with three repetitions (Table 2). Each Erlenmeyer flask was inoculated with 1 % (v/v) of three days *Bacillus* sp. strain BaH culture in MIM with 18 mM (*rac*)-MBA as described previously (Gord Noshahri et al. 2019). Incubation was performed in a rotary shaker (Infors-HT, Switzerland). The optimum condition was compared with initial condition (35 °C, pH 7.0 and 120 rpm).

**Table 2:** Design of experiments for the optimization of medium for biomass and ω-TA production in *Bacillus* sp. strain BaH, biomass production and AcPhe concentration were considered as a response

Run	A (°C)	B (rpm)	C (pH)	Biomass production (OD_600nm_)	AcPhe (mM)
1	33	160	7.7	0.18	0.04
2	33	160	8.8	0.6	0.03
3	33	160	7.7	0.25	0.04
4	28	120	7	0.27	0.67
5	33	160	7.7	0.19	0.03
6	33	216	7.7	0.09	0.01
7	28	200	8.5	0.39	0.01
8	40	160	7.7	1.61	0.03
9	33	160	7.7	0.17	0.19
10	33	100	7.7	0.56	0.01
11	33	160	6.7	3.24	2.76
12	38	200	7	5.65	3.29
13	33	160	7.7	1.92	0.66
14	26	160	7.7	0.22	0.02
15	38	120	8.5	0.94	0.01

Samples were taken daily for measuring the optical density at 600 nm (OD_600_), and pH value. After centrifugation (6000×*g*, 10 min) the supernatant of each sample was applied for measuring AcPhe concentration by HPLC.

### Biomass production optimization in the bioreactor

The best condition of *Bacillus* sp. strain BaH growth in shaking flask was optimized in Sixfors multiplex bench-top fermenter system (vessel volume 0.5 L; Infors AG, Switzerland). MIM (pH 7.0) was used as a culture medium and each fermenter was filled with 300 mL of medium. The fermenter was equipped with a pH sensor (Hamilton, Reno, NV, USA), optical dissolved oxygen sensor (Hamilton, Reno, NV, USA) and Pt-100 temperature sensor. Since antifoam addition can effect on cell growth (Routledge 2012), produced foam was transferred from exhausted cooler to plastic bag as a foam trap (Willenbacher et al. 2014). Fermentation temperature was set at 38 °C and all processes were run without pH regulation.

Stirrer speed and aeration were set up in the fermenter by one-factor-at-a-time method. In the mentioned method, to determine optimal level, only the effect of one factor was surveyed and other factors were kept constant. Then the optimized factor was considered as a basis. Each experiment was carried out in duplicate or triplicate. As depicted in Table 4, firstly, stirrer speed was surveyed in three levels of 200, 600 and 1200 rpm with 1.5 vvm aeration (Run1). At Run2, to determine proper agitation rate, the range of agitation speed was minimized and set around optimum of Run1. At the next step, aeration rate was evaluated in three levels of 0.5, 1 and 1.5 vvm with the best stirrer speed as Run3.

The inoculum was grown in a 100 mL in shaking flask in MIM medium (pH 7.0) and cultivated at 38 °C with 200 rpm in a rotary shaker (Infors-HT, Switzerland) for 2 days. The fermenters were run with 5 % (v/v) inoculum with initial optical density ~ 0.1. As a negative control, MIM medium containing AcPhe (3mM) was run in fermenter without any bacteria. Samples were taken four times a day. The samples were applied for analysis after centrifugation for 10 min at 6000×*g* with bench-top centrifuged (Eppendorf, Germany). The supernatants were used for quantification of AcPhe and glycerol.

At the time of sampling, DO and pH were recorded along with measurement of OD_600nm_. The cells from the sample with the highest amount of AcPhe were taken for ω-TA activity assay and measurement of kinetic parameters.

### Colorimetric ω-transaminase activity assay by native PAGE

Cell-free extraction of 3FCR was carried out according to Buß et al. (2018b) and applied as a positive control. Crude extract of *Bacillus* sp. strain BaH was obtained as described previously (Gord Noshahri et al. 2019). The concentration of protein was estimated with Roti®-Quant universal kit (Carl Roth, Karlsruhe, Germany). Glycerol was subsequently added to crude extracts obtained final concentration of 20 % (v/v) and extracts were stored at − 80 °C.

Polyacrylamide gel 12 % (v/v) was prepared based on Laemmli (1970) in the absence of any denaturant like SDS. Native sample buffer (62.5 mM Tris-HCl pH 6.8, glycerol 40 %, 0.01 % bromophenol blue) was added to each sample with ratio of 1:1. In each well 10 µL of sample (30 µg protein) without heating was loaded onto the gel. Samples were loaded in duplicate on both sides of gel in order to apply two kinds of staining. Electrophoresis was carried out in Laemmli running buffer without SDS, pH 8.0 at 4 °C with 150 V for 2 h. Afterwards the gel was divided into two sections containing samples and pre-stained protein marker (Roti®-Mark TRICOLOR, Carl Roth, Karlsruhe, Germany). Each gel section was transferred to different petri dishes. One gel section was stained for activity, the other section for localization of protein bands via Coomassie staining. Activity staining containing 50 mM HEPES buffer pH 7.5, 1 mM PLP, 5 mM OXD (amine donor), 7.5 mM pyruvate (amine acceptor) and 5 % DMSO was applied in one petri dish. The reaction was performed at 35 °C, 500 rpm for 30 min. Proteins in another section of gel were visualized by Coomassie Brilliant Blue R250 (Laemmli 1970).

### Kinetic parameters of BaH-ω-TA

Determination of kinetic constants was conducted by measuring AcPhe formation. Apparent *K*_m_ was investigated at varying concentrations of (*S*)-MBA and pyruvate in a Britton-Robinson buffer (pH 6) including 15 % (v/v) DMSO, 0.1 mM PLP, and 1 mg/mL crude extract in the total reaction volume of 250 µL: One substrate was varied in concentration and the other one was kept at a constant concentration. For calculating kinetic parameters of BaH-ω-TA for amine acceptor, pyruvate was added to reaction in the range of 0 to 70 mM with 70 mM (*S*)-MBA constant. For measuring kinetic parameters of BaH-ω-TA for amine donor, (*S*)-MBA was applied in a reaction between 0 and 60 mM with constant amount of 60mM pyruvate. The reactions were performed at 35 °C, 600 rpm for one hour. Subsequently, the reaction was stopped by heating at 95 °C for 5 min. The supernatant was separated by centrifugation (6000×*g*, 10 min) and produced AcPhe was detected by HPLC. Each reaction was conducted in triplicate. The apparent *K*_m_ for (*S*)-MBA and pyruvate was calculated based on Michaelis–Menten kinetics using non-linear regression method.

### **Quantification of AcPhe by HPLC**

The AcPhe concentration was measured via UV detection at 254 nm by mobile phase containing acetonitrile/water (50/50, v/v) at flow rate of 0.6 ml min^− 1^ (Gao et al. 2017) at room temperature using a C_18_ Hypersil- keystone column (250 × 4.6 mm 5 µ Hypersil). The injection volume was 1 µL. Agilent 1100 HPLC system (Santa Clara, CA, USA) was used for samples analysis.

### Quantification of glycerol by HPLC

The concentration of glycerol in the samples was analyzed with Agilent 1100 HPLC system (Santa Clara, CA, USA) with RI-detector system (G1362A RI Detector). Separation was carried out using LC column (Rezex TM ROA-organic acid H+ (8 %), 300 × 7.8 mm, with pre-column ROA-organic acid H+ (8 %) (50 × 7.8 mm), both Phenomenex, Germany) and a mobile phase consisting of 5 mM H_2_SO_4_ at a flow rate of 0.50 mL/min at 50 °C. The injection volume was set to 5 µL.

## Results

### Effect of cultivation medium on ω-TA induction

**Fig. 2:**
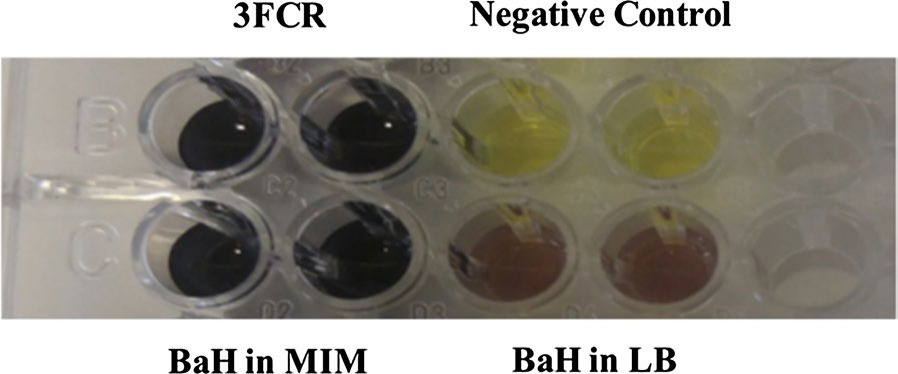
Effect of medium in induction of ω-TA. Cells grown in LB and MIM media with equal OD were applied in the reaction, including OXD and pyruvate as amine donor and amine acceptor, respectively, in HEPES buffer. 3FCR was used as a positive control. Reaction without cell was applied as a negative control

Inducible expression of ω-TA from *Bacillus* sp. strain BaH showed that enzyme production also happened without using (*rac*)-MBA in complex medium such as LB, although activity of ω-TA increased significantly in the presence of (*rac*)-MBA as a sole nitrogen source in the minimal medium (MIM) which was therefore preferred in the following. A comparison of ω-TA activity in whole cells is shown in Fig. 2. The color development of ω-TA containing crude extract of *Bacillus* sp. strain BaH after growth in MIM showed a significantly higher intensity than after growth in LB: An intensity level similar to the recombinantly overexpressed 3FCR was gained.

### Optimization of culture condition based on RSM

An experiment of 15 runs with three repetitions containing 5 central points was designed based on three factors (Table 2). The amount of biomass production (OD_600nm_) along with AcPhe production (deaminated form of MBA) was measured daily for 4 days (Table 2). There was a positive correlation between growth and AcPhe production. Therefore, the amount of biomass was chosen as response to analyze the effect of three factors on cell growth and ω-TA production with Design Expert 7.0. Based on 15 runs, the quadratic model was fitted to the response variable, and could be explained by the following equation where A, B and C represent temperature, agitation speed and pH, respectively:

$${\text{Biomass production }}({\text{OD}}_{600{\text{nm}}})=-3.85+1.72 A+0.21B-9.79 C+1.03E-0.03AB-0.38AC-0.036BC+0.01{A}^{2}+6.88E-0.05{B}^{2}+1.73{C}^{2}$$

The analyses of variance of regression for biomass production by *Bacillus* sp. *strain BaH* are summarized in Table 3. The *R*^*2*^ value was 0.9891, which indicates the accuracy of the model. The *P* value serves as a tool for checking the significance of each of the coefficients: If it is > 0.05 (not significant), the model fits well.

**Table 3:** Analysis of variance of calculated model for biomass production

Source	Sum of		Mean	F	p-value	
	Squares	df	Square	Value	Prob > F	
Model	32.07	9	3.56	50.58	0.0002	Significant
A-Temperature	0.61	1	0.61	8.70	0.0319	Significant
B-Agitation speed	0.20	1	0.20	2.79	0.1560	Not significant
C-pH	3.46	1	3.46	49.13	0.0009	Significant
AB	0.085	1	0.085	1.21	0.3209	Not significant
AC	4.10	1	4.10	58.15	0.0006	Significant
BC	2.29	1	2.29	32.49	0.0023	Significant
A^2^	1.38	1	1.38	19.55	0.0069	Significant
B^2^	0.10	1	0.10	1.43	0.2856	Not significant
C^2^	7.05	1	7.05	100.05	0.0002	Significant
Residual	0.35	5	0.070			
Lack of Fit	0.22	1	0.22	6.73	0.0605	Not significant
Pure Error	0.13	4	0.033			
Cor Total	32.42	14				

**Fig. 3:**
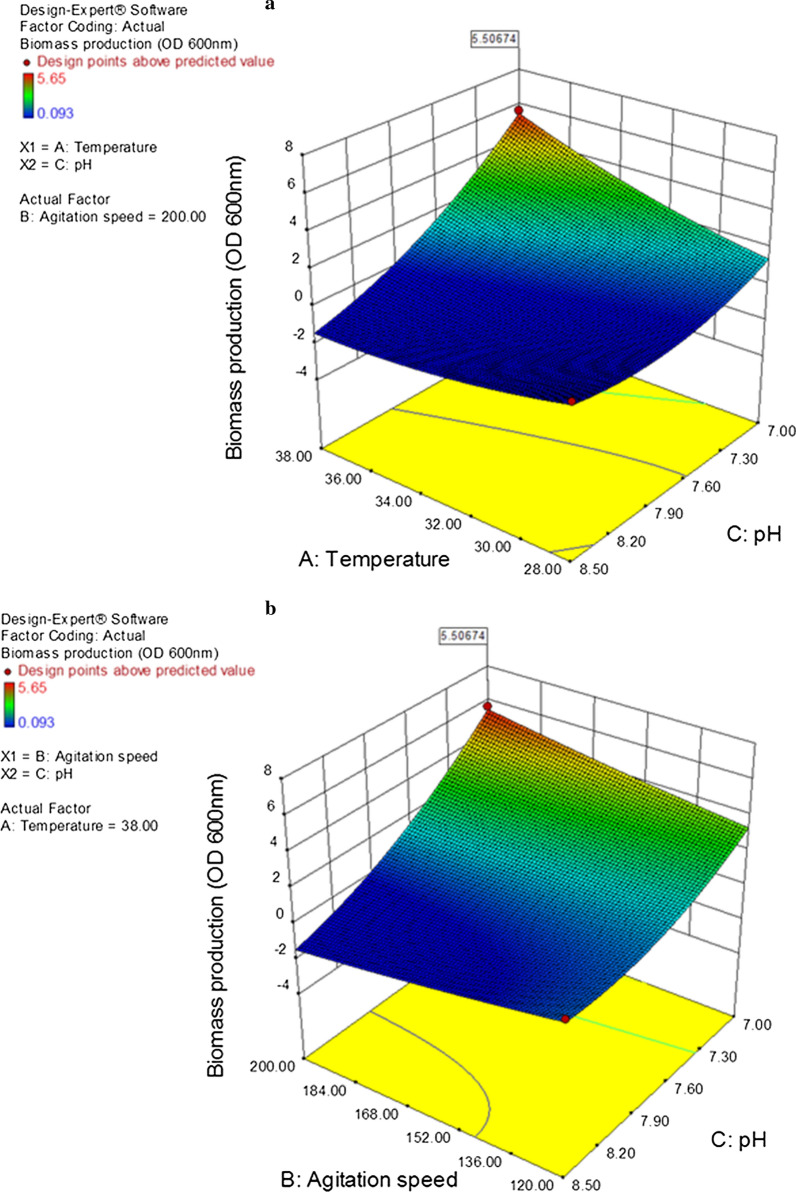
Response surface plot of Biomass production for *Bacillus* sp. strain BaH illustrates the interaction between two variables by keeping other one constant. **a** Interaction between temperature and pH while agitation speed is 200 rpm; **b** interaction between agitation speed and pH while temperature is 38 °C

In this case $A,\,C,\,AC,\,BC,\,{A}^{2},\,{ C}^{2}$ are significant model terms (p < 0.05) in biomass production while $B$ and $AB$ were not significant (p > 0.05). Figure 3 presents response surface plot of interaction between two variables while third one kept constant. According to the plot the interaction between pH and temperature (Fig. 3a) and pH and agitation speed (Fig. 3b) are significant. The optimum condition of variables can be understood easily from the plot.

According to the model, a temperature of 38 °C, pH 7.0 and agitation with 200 rpm were determined as optimum conditions. Applying optimum values led to a final OD_600nm_ of 5.4 which agreed well with predicted value (OD_600nm_ = 5.5). In addition, Maximum AcPhe concentration in optimum condition was determined 3.4 mM.

Comparing with initial condition with OD_600nm _∼ 2.9, biomass production was estimated to be around 1.9 times enhanced. As a result of growth optimization and using MBA as nitrogen source, AcPhe concentration emerged 2.6 times higher than at the initial condition with 1.3 mM AcPhe.

The highest AcPhe production in optimum condition was measured after 72 h which was immediately before the culture reached the stationary phase.

### Batch culture of *Bacillus* sp. strain BaH in bioreactor

The optimum condition in shaking flask including temperature 38 °C and pH 7.0 were transferred to the Sixfors bioreactor system, while stirrer speed and aeration rate were developed in the bioreactor (Additional file 1: Figure S1). Table 4 shows the optimization results. The evaluation of both biomass and AcPhe concentration indicated 0.5 vvm aeration with 600 rpm stirrer speed as best conditions. Stirrer speed showed direct effect on biomass production: Both less and more than 600 rpm had negative effect on the amount of biomass produced. All bioreactors shared almost the same amount of biomass, optical density at 600 nm was around 5 in all reactors, at different aeration rate levels with agitation of 600 rpm. However, AcPhe concentration dropped with increasing oxygen dissolved (DO) in culture.

**Table 4:** The design and result of one-factor-at-time for optimization of biomass production in bioreactor

Run	Variable factor	Constant factor	Fermenters [Absorbance in 600nm and AcPhe concentration mM (in parentheses)]					
			F1	F2	F3	F4	F5	F6
1	Stirrer speed (rpm)	Aeration (1.5 vvm)	200 rpm (1.8, 0.25 mM)		600 rpm (5.2, 1.9 mM)		1200 rpm ( 2.1, 0.3 mM)	
2			400 rpm (2.4, 1.7 mM)		600 rpm (5.1, 2.1 mM)		800 rpm (4.7, 1.4 mM)	
3	Aeration (vvm)	Stirrer speed (600 rpm)	0.5 vvm (5.3, 3.5 mM)		1 vvm (5.2, 2.6 mM)		1.5 vvm (5.4, 2.3 mM)	

**Fig. 4:**
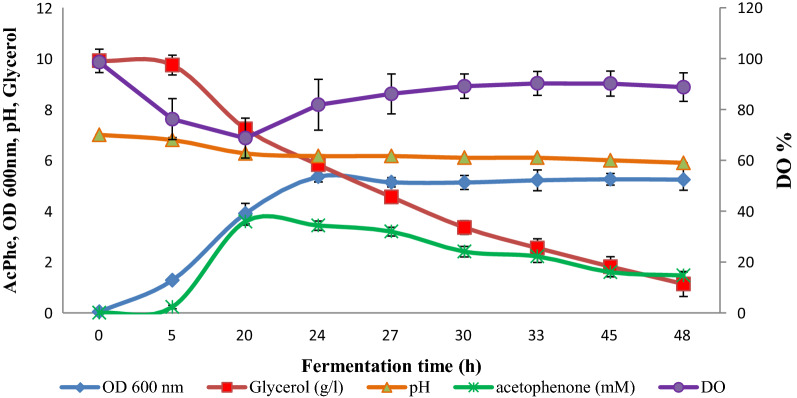
Fermentation process in batch culture of *Bacillus* sp. strain BaH in Sixfors bioreactor. Error bars represented standard deviation

The fermentation process of *Bacillus* sp. strain BaH under optimum condition (600 rpm, 0.5 vvm) is depicted in Fig. 4. As inoculum medium is similar with fermentation medium, no lag phase was recorded. Maximal growth was obtained 24 h after inoculation with optical density of 5.3. Detection of ω-TA activity by monitoring formation of AcPhe gave a maximum amount of 3.5 mM which was produced after 20 h. A coincidence was observed between dissolved oxygen (DO) depletion and AcPhe decline: A drop in DO was accompanied by a gradual decrease in AcPhe and glycerol concentration. DO declines with increasing OD, growth rate decreases and DO rises again after 20 h.

### ω-TA activity assay on native gel

Crude extracts of the *Bacillus* sp. strain BaH from the fermenter and a recombinantly expressed ω-TA (3FCR) in shaking flask were subjected to native PAGE and subsequently stained with OXD assay. Ten minutes after starting the staining reaction, a dark brown precipitation was observed which indicated an ω-TA band.

**Fig. 5:**
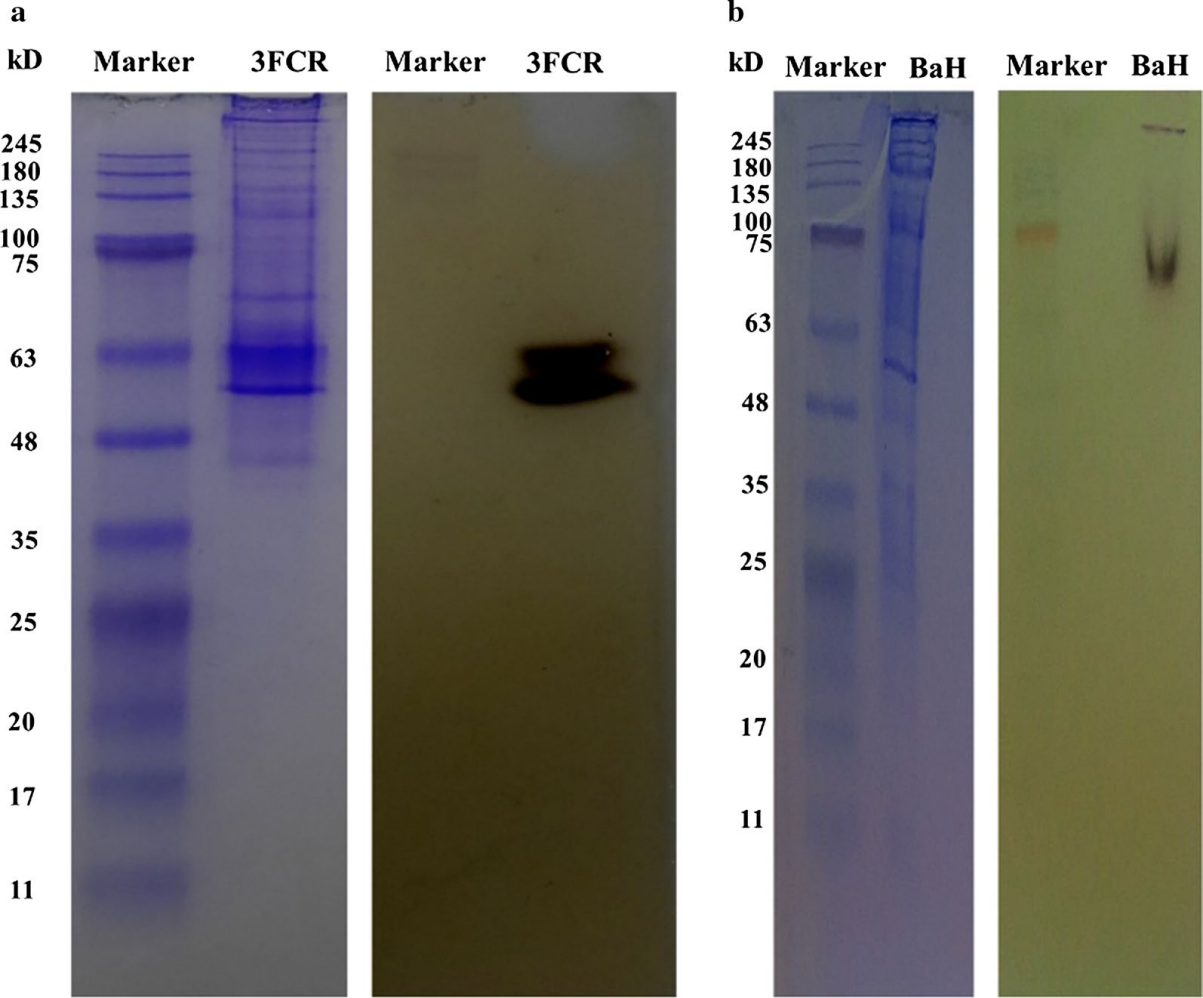
Colorimetric assay for localization of ω-TAs on native polyacrylamide gel. Crude extracts of **a** 3FCR, overexpressed in *E. coli*. **b** *Bacillus* sp. strain BaH, each gel from left to right, containing Coomassie brilliant blue staining (as described in method section) and activity staining assay was carried out in 50 mM HEPES buffer, pH 7.5 with OXD (5 mM), pyruvate (7.5 mM) DMSO (5 %) and PLP (1 mM). The numbers indicating the protein marker bands resemble the order of the bands when used for SDS-PAGE according to the manufacturer’s manual and cannot be used for molecular weight determination in native gels. However, we used this pre-stained marker to demonstrate the color development and the specific staining of the ω-TA band

Significant development of a dark precipitate was demonstrated with a crude extract of *Bacillus* sp. strain BaH (Fig. 5b). As a negative control, a conventional staining using Coomassie brilliant blue was performed to show that many other protein bands were present in the crude extract that were not stained with the OXD assay. As a positive control, crude extract of the ω-TA with PDB code 3FCR overexpressed in *E. coli* led to a strong reaction and a distinct black band. Again, a Coomassie staining demonstrated the exclusive staining of the overexpressed ω-TA band in the OXD assay whereas a plethora of other protein bands did not show any visible reaction (Fig. 5a).

### Kinetic parameters of BaH-ω-TA

Kinetic constants were estimated by non-linear regression based on Michaelis-Menten kinetics. The apparent *K*_m_ of BaH-ω-TA value for (*S*)-MBA and pyruvate were calculated 2.6 mM and 17.3 mM, respectively (Additional file 1: Figure S2).

## Discussion

Although ω-TAs received considerable attention recently and are used in industrial reactions both as isolated enzymes and as whole-cell catalysts, to the best of our knowledge there is no report to optimize ω-TAs production in wild-type strains as applied for industrial catalysts (Almyasheva et al. 2018). Determining the influence of culture condition on biomass and ω-TA production poses a major challenge for industrial application of enzymes which cannot be easily expressed in established production strains, e.g. enzymes from extremophiles.

The aim of this study was to achieve growth optimization and improved enzyme induction of *Bacillus* sp. strain BaH in shaking flask and bioreactor in a small scale in order to produce ω-TA and identify kinetic parameters of this enzyme. In accordance with Shin et al. (2001), ω-TA activity is considerably increased in minimal media in comparison to LB medium depending on particular nitrogen sources offered in the medium.

Therefore, minimal medium containing MBA as a sole nitrogen source was used in this study to induce ω-TA production during the growth of *Bacillus* sp. strain BaH. ω-TA activity during cultivation was monitored by measuring formation of AcPhe (deaminated form of MBA). Cell growth is affected by enzyme production depending on MBA as a sole nitrogen source. Thus, microbial growth accelerates with increasing intracellular ω-TA activity. Consequently, cells should be in their exponential growth phase with high cell density to extract and purify ω-TA. Shin et al. harvested *Vibrio fluvialis* cell in the late exponential phase to separate ω-TAs of bacteria (Shin and Kim 2001).

RSM software was applied for optimization of culture condition in shake flask. It was found that the produced biomass under optimum condition (38 °C, pH 7.0 and 200 rpm) was 1.9 times higher than under initial condition (30 °C, pH 7.0 and 120 rpm). The optimum condition was scaled up in bench-top bioreactor with changing agitation speed to 600 rpm. Notably, scale up cultivation from shaking flask to bioreactor share similar biomass and AcPhe concentration, reaching approximately 5.3 and 3.4 mM, respectively, which indicated a good determination of parameters involved in growth in the bioreactor.

During fermentation in bioreactor, it was observed that AcPhe concentration decreased in the late exponential phase. A control experiment with AcPhe was run in parallel to exclude evaporation of this compound. It was demonstrated that AcPhe depletion via evaporation in bioreactor was not notable (data not shown). AcPhe decreasing during fermentation was also reported by Buß et al. (2018a), who discussed the possibility of bacterial metabolization of AcPhe. Besides, it was shown that alcohol dehydrogenases (ADH) from *Lactobacillus kefir* accepts AcPhe and converts it to (*R*)-alcohol (Yun et al. 2003). Degradation of 4-hydroxyacetophenone and conversion to phenyl-acetate in the presence of molecular oxygen by Baeyer-Villiger monooxygenases in *Pseudomonas putida* JD1 is reported by Rehdorf et al. (2009). Moreover, microbial degradation of AcPhe catalyzed by oxygenases has been approved in *Arthrobacter* sp., *Micrococcus* sp. (Havel and Reineke 1993) and *Alcaligenes* sp. (Higson and Focht 1990). Noticeably, the former strain showed ω-TA activity (Iwasaki et al. 2006; Päiviö and Kanerva 2013) and its ω-TA mutant (ATA-117) is produced commercially with collaboration of Codexis and Merck (Savile et al. 2010). The enzymatic degradation of AcPhe would be a potential way to overcome ω-TAs inhibition by their ketone product.

As an additional aspect of the study, we present an efficient colorimetric assay to visualize ω-TAs in crude extracts on acrylamide gel by using *ortho*-xylylenediamine (OXD) as amine donor. The application of the OXD assay established by Green et al. (2014) is thereby expanded from high-throughput activity screenings and colony-based screenings of heterologously expressed mutants to the protein level to directly identify the desired enzyme in wild-type strains. Results of separate studies clearly demonstrated that OXD is a specific diamine substrate for ω-TA (Green et al. 2014; Kelly et al. 2017). Therefore, we applied this substrate for a colorimetric assay on acrylamide gel. The strategy explained is a simple and fast method for localization of ω-TA directly in crude extract on polyacrylamide gel in order to enable identification and further sequencing of the enzyme if a sufficient protein amount and purity is given. Conducting Coomassie blue staining indicates that this assay appears sensitive without any background color and a need for removing by-product. In particular, several α-transaminases (α-TA) are constitutively expressed enzymes in *Bacillus* as well as in *Escherichia* as in every living cell (Dold et al. 2016) and should have been stained with an unspecific staining method. So the OXD assay appears to be specific for ω-TAs.

While the original OXD assay was established for screening large substrate panels or libraries of ω-TA variants to facilitate the development of the next generation of ω-TA biocatalysts by directed evolution (Green and Turner 2016), our modification may prove beneficial for a fast screening for novel ω-TAs from natural isolate screenings. In particular, the direct isolation of the desired ω-TA protein band from the gel for further investigations should be possible if a sufficient amount of enzyme is applied. Kim et al. (2007) reported native gel activity staining for rapid partial purification of so-called β-transaminase by using a nitroblue tetrazolium colorimetric assay. The resulting single purple band of protein was used for amino acid sequencing. In this method, removing alanine produced during the reaction and regeneration of pyruvate by using alanine dehydrogenase plus NAD^+^ as a cofactor are essential parts of color development which makes this approach rather complex.

Development of an enzymatic reaction involves determination of kinetic constants (Villegas-Torres et al. 2018). We calculated the Michaelis–Menten constants as 2.6 mM and 17.3 mM for (*S*)-MBA and pyruvate, respectively, which represented the good affinity of (*S*)-MBA to the enzyme. Jiang et al. (2014) calculated the apparent *K*_m_ of an ω-TA from *Burkholderia vietnamiensis* for (*S*)*-*MBA and pyruvate as 14.67 mM and 1.65 mM, respectively. On the other hand, the apparent *K*_m_ of an ω-TA from *Alcaligenes denitrificans* toward pyruvate was reported 11 mM in the presence of 10mM l-β-amino-*n*-butyric acid (Yun et al. 2004).

## Supplementary Information

**Additional file 1: Figure S1.** Batch culture of* Bacillus* sp. strain BaH in Sixfors multiplex bench-top fermenter system. Plastic bags were used as a foam trap. **Figure S2.** Determination of kinetic constant of BaH-ω-TA [1 mg/ml crude extract in the total reaction volume of 250 µL, see method section] for (*S*)-MBA (red line) in 0-60mM concentration in the presence of 60mM pyruvate and for pyruvate (gray line) in 0-70 mM concentration in the presence of 70 mM (*S*)-MBA.

## Data Availability

All bacterial strains and enzymes investigated in this study are available from the authors.

## Abbreviations

ω-TA: ω-Transaminase
BaH-ω-TA: ω-TA of *Bacillus *sp. strain BaH
DMSO: Dimethyl sulfoxide
MBA: Methylbenzylamine
OXD: *ortho*-Xylylenediamine
DO: Dissolved oxygen
OD: Optical density
AcPhe: Acetophenone
