# Supplementary material for: Growth optimization and identification of an ω-transaminase by a novel native PAGE activity staining method in a Bacillus sp. strain BaH isolated from Iranian soil
Source: AMB Express. 2021 Mar 23;11:46. doi: 10.1186/s13568-021-01207-7 (PMC7988029; doi:10.1186/s13568-021-01207-7)
Supplement: Supplementary file 1 — Additional file 1: Figure S1. Batch culture of Bacillus sp. strain BaH in Sixfors multiplex bench-top fermenter system. Plastic bags were used as a foam trap. Figure S2. Determination of kinetic constant of BaH-ω-TA [1 mg/ml crude extract in the total reaction volume of 250 µL, see method section] for (S)-MBA (red line) in 0-60mM concentration in the presence of 60mM pyruvate and for pyruvate (gray line) in 0-70 mM concentration in the presence of 70 mM (S)-MBA. [file 13568_2021_1207_MOESM1_ESM.docx]

**Growth optimization and identification of an ω-transaminase by a novel native PAGE activity staining method in a *Bacillus* sp. strain BaH isolated from Iranian soil**

Najme Gord Noshahri ^1, 2*^, Jamshid Fooladi^1^, Ulrike Engel^2^, Delphine Muller^2^, Michaela Kugel^2^, Pascal Gorenflo^2^, Christoph Syldatk^2^, and Jens Rudat^2, *^

^1^ Department of Biotechnology, Faculty of Biology Science, Alzahra University, Tehran, Iran

^2^ Karlsruhe Institute of Technology (KIT), BLT 2 Technical Biology, Fritz-Haber-Weg 4, Karlsruhe, Germany

Correspondence: jens.rudat@kit.edu, n.noshahri@alzahra.ac.ir


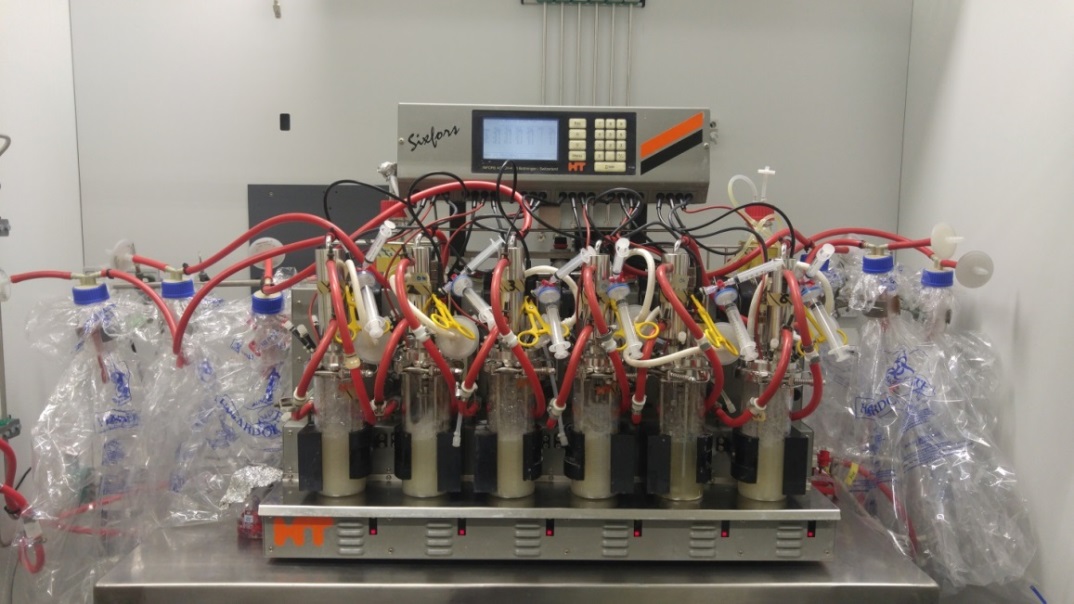


**Figure S1** Batch culture of *Bacillus* sp. strain BaH in Sixfors multiplex bench-top fermenter system. Plastic bags were used as a foam trap.


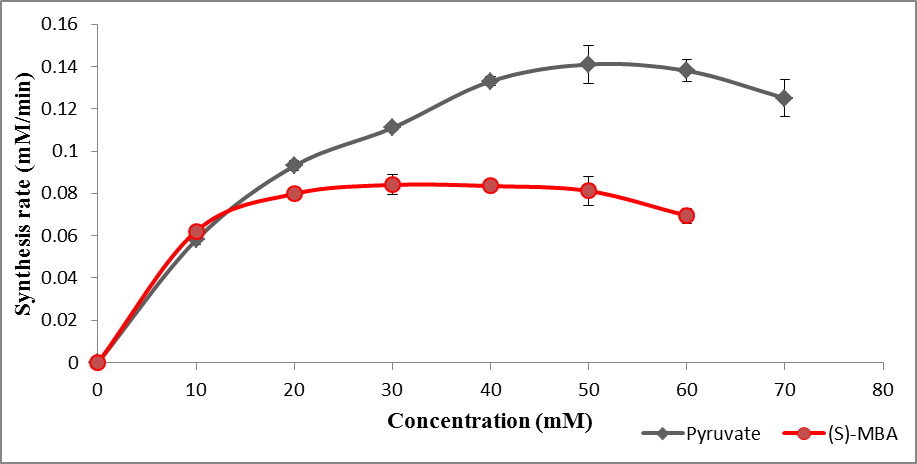


**Figure S2** Determination of kinetic constant of BaH-ω-TA [1 mg/ml crude extract in the total reaction volume of 250 µL, see method section] for *(S)*-MBA (red line) in 0-60mM concentration in the presence of 60mM pyruvate and for pyruvate (gray line) in 0-70 mM concentration in the presence of 70 mM *(S)-*MBA.
